# Multiple blocks in the engagement of oxidative phosphorylation in putative ovarian cancer stem cells: implication for maintenance therapy with glycolysis inhibitors

**DOI:** 10.18632/oncotarget.2367

**Published:** 2014-08-19

**Authors:** Ayesha B. Alvero, Michele K. Montagna, Natalia J. Sumi, Won Duk Joo, Emma Graham, Gil Mor

**Affiliations:** ^1^ Department of Obstetrics, Gynecology and Reproductive Sciences, Yale University School of Medicine, New Haven, CT, USA

**Keywords:** ovarian cancer, cancer stem cells, oxidative phosphorylation, Warburg effect, maintenance treatment

## Abstract

Survival rate in ovarian cancer has not improved since chemotherapy was introduced a few decades ago. The dismal prognosis is mostly due to disease recurrence where majority of the patients succumb to the disease. The demonstration that tumors are comprised of subfractions of cancer cells displaying heterogeneity in stemness potential, chemoresistance, and tumor repair capacity suggests that recurrence may be driven by the chemoresistant cancer stem cells. Thus to improve patient survival, novel therapies should eradicate this cancer cell population. We show that in contrast to the more differentiated ovarian cancer cells, the putative CD44+/MyD88+ ovarian cancer stem cells express lower levels of pyruvate dehydrogenase, Cox–I, Cox-II, and Cox–IV, and higher levels of UCP2. Together, this molecular phenotype establishes a bioenergetic profile that prefers the use of glycolysis over oxidative phosphorylation to generate ATP. This bioenergetic profile is conserved *in vivo* and therefore a maintenance regimen of 2-deoxyglucose administered after Paclitaxel treatment is able to delay the progression of recurrent tumors and decrease tumor burden in mice. Our findings strongly suggest the value of maintenance with glycolysis inhibitors with the goal of improving survival in ovarian cancer patients.

## INTRODUCTION

Epithelial ovarian cancer (EOC) accounts for the greatest number of deaths from gynecologic malignancies [1-3]. A newly diagnosed patient commonly presents with advanced-stage disease but usually undergoes remission after initial surgical debulking and first-line chemotherapy. Unfortunately, in spite of the initial response to treatment, almost all patients recur within 2 to 5 years [4]. At recurrence, the development of resistance to most of the currently available agents limits the efficacy of second round chemotherapy. Thus the five-year survival rate for EOC remains very low at about 30% [4]. Therefore, to improve survival, it is necessary to identify novel treatment modalities that can prevent recurrence or target chemoresistant recurrent disease.

Chemotherapy for ovarian cancer was initiated in the 1970s and data from multiple trials has supported the advantage of using the combination of platinum (most often carboplatin) and taxane (most often paclitaxel) compounds [5-7]. Although the mechanisms of action of chemotherapy agents are diverse, most exploit the difference in the proliferation rate between normal cells and cancer cells. Highly proliferating cancer cells are more sensitive to the effects of DNA damage and microtubule stabilization, the actions of platinum and taxane compounds, respectively. However, despite advances in the understanding of how these compounds induce cell death and how chemoresistance develops at the molecular level, this knowledge has not translated into better survival for patients.

In the past decade, numerous studies have demonstrated the heterogeneity of cancer cells that comprise the tumors. A specific cancer cell population, the cancer stem cells, has been shown to be responsible for tumor-initiation, progression, recurrence and chemoresistance [8-18]. Compared to their differentiated counterparts, cancer stem cells isolated from various cancers have consistently been shown as inherently and extremely resistant to currently available chemotherapy agents. It is hypothesized that cancer stem cells left after surgery can survive chemotherapy and are able to re-establish the tumor leading to recurrence [17, 19]. In EOC, we and others have shown that CD44+/MyD88+ epithelial ovarian cancer stem cells (EOC stem cells) possess tumor-initiating properties and exhibit diverse resistance to a wide-range of chemotherapy agents including, but not limited to, platinums and taxanes [20-26]. Interestingly, CD44+/MyD88+ EOC stem cells do not proliferate as rapidly as differentiated cancer cells [20]. This may be one of the myriad reasons why this cancer cell population is resistant to chemotherapy.

In addition to the proliferation rate, another main difference between cancer cells and normal cells is the predilection of cancer cells, even in normoxic conditions, to utilize the less efficient glycolysis pathway over the more efficient mitochondrial oxidative phosphorylation (OXPHOS) to generate ATP (Warburg effect) [27, 28]. Indeed, it has been demonstrated for decades that compared to normal cells, cancer cells have enhanced glucose uptake, high lactic acid production, and almost absent mitochondrial respiration even when oxygen levels are sufficient [29, 30]. Enhanced glucose uptake in cancer cells have been demonstrated to fulfill the ATP and NADPH requirements of fast-dividing cells [31]. It is however not clear if the Warburg effect also occurs in cancer stem cells. This is extremely important, since this would suggest that, whereas classical chemotherapy agents are only effective against the differentiated cancer cells, compounds inhibiting glycolysis may target both differentiated cancer cells as well as the more undifferentiated and chemoresistant cancer stem cells. Therefore glycolysis inhibitors may be used in combination with the standard of care chemotherapy agents. Such regimen would target the two known cancer cell populations, possibly preventing recurrence and improving patient survival.

The aim of this study is to determine the unique bioenergetic requirements of CD44+/MyD88+ EOC stem cells. Towards this goal we compared the requirement for glycolysis and mitochondrial OXPHOS in the generation of ATP in clones of EOC stem cells and their differentiated counterpart (CD44-/MyD88- EOC cells). Our results show that in the chemoresistant CD44+/MyD88+ EOC stem cells, baseline ATP is mainly produced from the glycolysis pathway and moreover the generation of ATP from OXPHOS is blocked at multiple levels. Therefore, under glucose limiting conditions and even in the presence of OXPHOS substrates, EOC stem cells are unable to efficiently and promptly engage OXPHOS leading to subsequent loss of ATP and consequently cell death. We also provide a proof of concept demonstrated in an intra-peritoneal (i.p.) ovarian cancer xenograft model, which shows that glycolysis inhibitors are able to delay recurrence and inhibit progression of recurrent disease. Our study highlights the value of a maintenance regimen with glycolysis inhibitors to improve survival in ovarian cancer patients.

## RESULTS

### CD44+/MyD88+ EOC stem cells require glucose for survival

**Figure 1:**
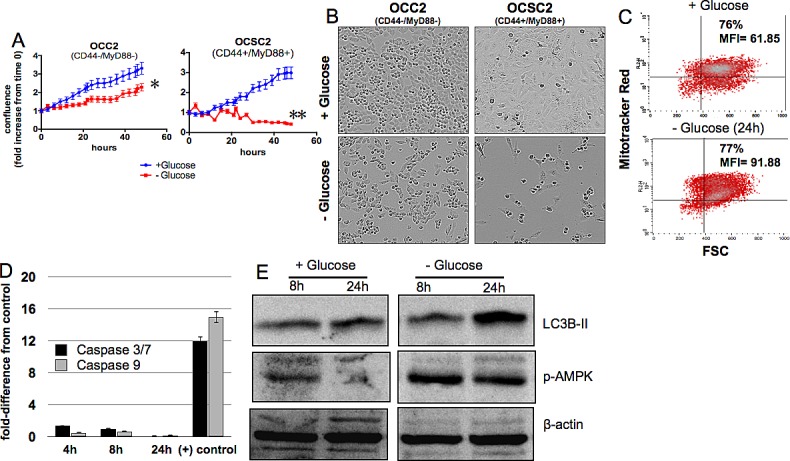
CD44+/MyD88+ EOC stem cells require glucose for survival CD44+/MyD88+ EOC stem cells and CD44-/MyD88- EOC cells were grown in glucose-enriched or glucose-free conditions as described in the Methods section. (A) Growth rate and (B) cellular morphology were determined using the Incucyte kinetic live cell imaging system. (C) Mitochondrial membrane potential was quantified by flow cytometry using Mitotracker Red fluorescence, p> 0.5, not significant. (D) Caspase activity was measured using Caspase- 3/7 and 9 Glo assays with the positive control described in the Methods section, p> 0.5, not significant. (E) Autophagy-induced cleavage of LC3B and activation of AMPK were determined by Western blot analysis. * p = 0.0008; ** p < 0.0001.

To determine the response of the two subtypes of ovarian cancer cells to glucose deprivation, we cultured the clones described in the Methods section in the presence or absence of glucose and quantified the growth rate by measuring culture confluence. We saw a significant reduction in the growth rate of CD44-/MyD88- EOC cells under glucose-free condition compared to glucose-enriched condition (Fig. 1A). Whereas the doubling-time for the clones in glucose-enriched media is ~18h, the doubling-time in glucose-free condition was ~40h. The decrease in growth rate was however, not accompanied by cell death. Analysis of cellular morphology showed that in glucose-free condition, CD44-/MyD88- EOC cells remained viable (Fig. 1B), albeit with a slower proliferation rate. On the other hand, we observed significant cell death in the CD44+/MyD88+ EOC stem cells 12h after the addition of glucose-free media (Fig. 1A,B). Interestingly, further studies showed that cell death in these cells is not associated with typical apoptotic markers. We did not observe loss of mitochondrial membrane potential (Fig. 1C) nor activation of caspases (Fig. 1D). Instead, we observed the occurrence of autophagy as evidenced by increase in LC3B-II (Fig. 1E). These results indicate that whereas CD44-/MyD88- EOC cells require glucose to sustain their baseline proliferation rate, CD44+/MyD88+ EOC stem cells require glucose for survival and therefore suggest dependence to glucose.

### CD44+/MyD88+ EOC stem cells have a more glycolytic phenotype

**Figure 2:**
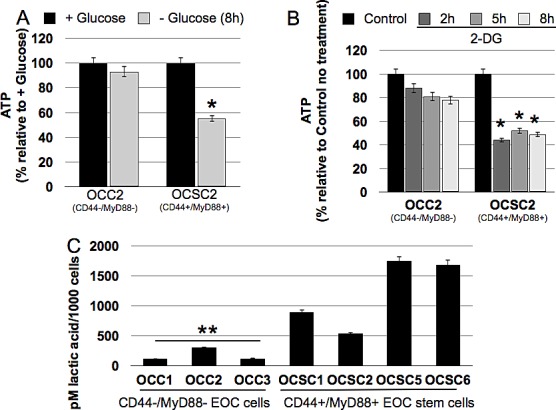
CD44+/MyD88+ EOC stem cells have a glycolytic phenotype (A) Cells were grown in glucose-enriched or glucose-free conditions or (B) treated with 20 mM 2-DG and ATP levels quantified using Celltiter Glo. (C) Lactic acid was quantified as described in the Methods section using cell-free supernatants. * p = 0.001 compared to + Glucose or Control no treatment; ** p = 0.03, OCC vs OCSC.

The demonstration that CD44+/MyD88+ EOC stem cells undergo cell death when glucose is not available suggests that these cells significantly rely on the glycolysis pathway for their energy needs. To confirm this, we quantified ATP in all the clones in the presence or absence of glucose. Our results showed that CD44-/MyD88- EOC clones can maintain ATP in glucose-free media (Fig. 2A). In contrast, we observed a significant decrease in ATP levels in CD44+/MyD88+ EOC stem cell clones when grown in the absence of glucose (Fig. 2A). The decrease in ATP may be associated with the observed increase in phosphorylated AMPK (pAMPK) (Fig. 1E), and probably contributes to the observed autophagic cell death in the EOC stem cells. The glycolysis inhibitor 2-deoxyglucose (2-DG) had similar effect on the clones. ATP levels were marginally decreased in CD44-/MyD88- EOC clones but significantly decreased in the CD44+/MyD88+ EOC stem cell clones treated with 2-DG (Fig. 2B). These results demonstrate that in the EOC stem cells baseline ATP is maintained primarily by the glycolysis pathway.

Glucose-avid cells are known to have higher levels of glucose transporters and enzymes involved in the glycolysis pathway. Upregulation of these proteins have been the classical method to demonstrate a glycolytic phenotype. We have previously performed gene expression microarray analysis comparing CD44+/MyD88+ EOC stem cells and CD44-/MyD88- EOC cells [20]. Using the data generated, we performed pathway analysis and surprisingly did not observe any significant difference in the key bioenergetic pathways tested, at least at the message level (data not shown). However, functional analysis such as quantification of lactic acid secretion showed that CD44+/MyD88+ EOC stem clones (OCSC1, OCSC2, OCSC5, OCSC6) produce higher levels of lactic acid per cell compared to CD44-/MyD88- EOC clones (OCC1, OCC2, OCC3) (Fig. 2C). Thus, although we did not see a difference at the mRNA level, the lactic acid quantification assay demonstrate that indeed CD44+/MyD88+ EOC stem cells are more glycolytic than CD44-/MyD88- EOC cells.

### CD44+/MyD88+ EOC stem cells cannot engage OXPHOS

**Figure 3:**
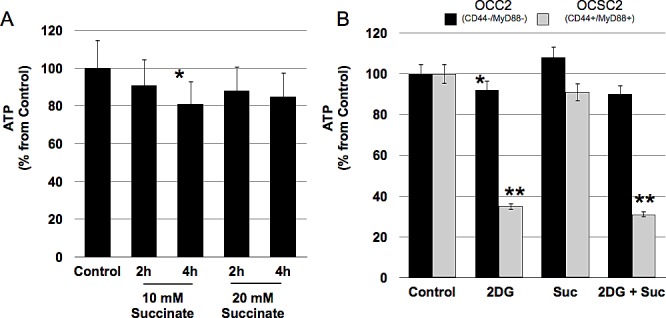
CD44+/MyD88+ EOC stem cells cannot switch to oxidative phosphorylation when glucose levels are limiting (A) EOC stem cells cells were treated with 20 mM Succinate to stimulate oxidative phosphorylation and ATP levels quantified as above. (B) Cells were pre-treated with 20 mM 2-DG for 2h then co-treated with 20 mM Succinate for another 2h prior to measuring ATP. * p > 0.05, not significant; ** p= 0.0001 compared to Control.

Inability to maintain ATP levels in the absence of glucose or in the presence of glycolysis inhibitors suggest that EOC stem cells can not switch to mitochondrial OXPHOS. OXPHOS occurs within the electron transport chain (ETC) and results from the entry of reducing equivalents such NADH, FADH2 or succinate generated in the Citric Acid Cycle into either Complex I or Complex II of the ETC. Transfer of electrons from the reducing equivalents to the different components of the ETC leads to the generation of a proton gradient, which is used as energy to phosphorylate ADP and generate ATP. To determine the ability of EOC stem cells to generate ATP by OXPHOS we treated the clones with succinate. CD44+/MyD88+ EOC stem cell clones did not show an increase in ATP when treated with succinate (Fig. 3A). In addition, succinate was not able to rescue ATP levels in the EOC stem cells treated with 2DG (Fig. 3B). In contrast, an initial increase in ATP was observed in CD44-/MyD88- EOC cell clones treated with succinate although it was not sustained through time (Fig. 3B and data not shown). Taken together, these results show that EOC stem cells cannot switch to mitochondrial OXPHOS to generate ATP under glucose-limiting conditions and even when reducing equivalents are available.

### EOC stem cells possess more mitochondria but are not purposed for ATP production

**Figure 4:**
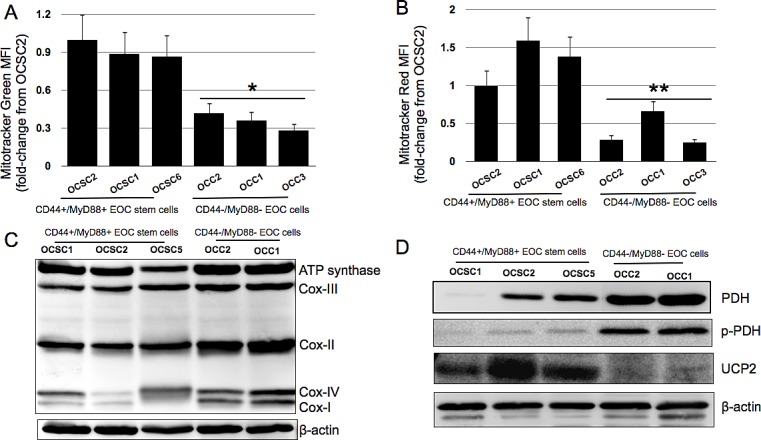
Mitochondrial phenotype of the CD44+/MyD88+ EOC stem cells (A), (B) Clones were labeled with Mitotracker Green FM or Mitotracker Red CMXRos and analyzed by flow cytometry. Data shown are normalized MFI values against OCSC2; (C) Western blot analysis of the different components of the electron transport chain and (D) total and phosphorylated pyruvate dehydrogenase and UCP2. * p = 0.0006, OCC vs OCSC; ** p = 0.013, OCC vs OCSC.

Earlier studies looking into the molecular foundation for the Warburg effect suggests that cancer cells rely on glycolysis for its ATP needs even in normoxic conditions due to defective mitochondria [32]. However, mutations or permanent mitochondrial defects cannot explain the dependence of CD44+/MyD88+ EOC stem cells on glucose since upon differentiation to CD44-/MyD88- EOC cells [20] this dependence is reduced. Thus, we characterized the mitochondria of the EOC stem cell clones and compared them with their differentiated counterpart. Using Mitotracker Green and Mitotracker Red, we quantified mitochondrial mass and mitochondrial membrane potential (MMP), respectively. Flow cytometry analysis demonstrate that compared to the CD44-/MyD88- EOC cell clones, CD44+/MyD88+ EOC stem cell clones possess more mitochondria. Mean fluorescent intensity (MFI) from Mitotracker green is two-fold higher in OCSC1, OCSC2, and OCSC6 compared to OCC1, OCC2, and OCC3 (Fig. 4A). With respect to MMP, which is a reflection of the ability of the ETC to generate a proton gradient, Mitotracker Red MFIs for CD44+/MyD88+ EOC stem cell clones are likewise higher compared to CD44-/MyD88- EOC cell clones (Fig. 4B). Therefore, the dependence of EOC stem cells to glucose and its inability to generate ATP by OXPHOS cannot be attributed to inferior levels of mitochondria nor inability to sustain optimal proton gradient.

We then characterize the mitochondrial components that are directly related to ATP production. We focused our attention on: 1) the components of the ETC; 2) pyruvate dehydrogenase (PDH), which is a key enzyme in shifting pyruvate towards the mitochondrial Citric Acid Cycle and therefore away from glycolyis and; 3) uncoupling protein 2 (UCP2), an anion/proton carrier that diverts the proton gradient away from ATP production towards the generation of heat. Western blot analysis of the different components of the ETC show that although CD44+/MyD88+ EOC stem cells possess more mitochondria than CD44-/MyD88- EOC cells, EOC stem cells have lower levels of Complex I, Complex II, and Complex IV (Fig. 4C). In addition, PDH is undetectable in OCSC2 and consistently lower in the EOC stem cell clones compared to their differentiated counterpart (Fig. 4D). This suggests that in the EOC stem cells, pyruvate is unable to enter the Citric acid cycle and therefore the generation of ATP from this molecule can only occur by conversion to lactic acid in the cytoplasm. Interestingly, in the differentiated CD44-/MyD88- EOC cells, although PDH is highly expressed, it mostly occurs in the phosphorylated or inactive state (Fig. 4D). This shows that PDH activity is regulated by different mechanisms in the two subtypes of EOC cells. Although the endpoint is inhibition, PDH activity is regulated in the CD44+/MyD88+ EOC stem cells by down-regulated expression, while in the differentiated CD44-/MyD88- EOC cells it is regulated by inactivation/phosphorylation. Finally, UCP2 levels are highly upregulated in the EOC stem cells (Fig. 4D) further averting ATP production from the established proton gradient. Taken together with the data showing that EOC stem cells cannot sustain ATP levels under glucose-limiting conditions, these results suggest that in these chemoresistant cells, which possess a significant amount of the organelle, mitochondrial function is not primarily for the generation of ATP.

### Oxidative phosphorylation is dispensable for EOC stem cell survival

**Figure 5:**
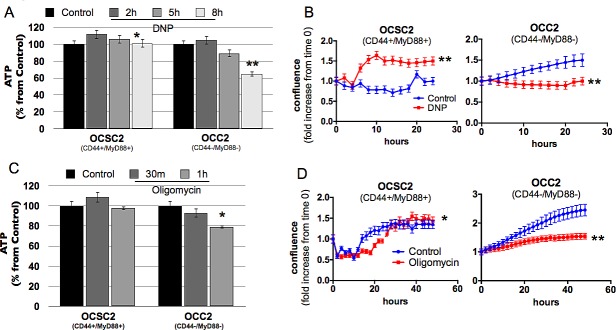
Oxidative phosphorylation is dispensable in the survival of CD44+/MyD88+ EOC stem cells Cells were treated at different time-points with 1 mM DNP (A,B) and 5 μM Oligomycin (C,D). ATP levels (A,C) were quantified by Celltiter Glo and growth rate (B,D) determined by Incucyte. * p > 0.99, not significant; ** p < 0.0001 compared to Control.

To conclusively show that OXPHOS is dispensable in the CD44+/MyD88+ EOC stem cells, we treated the clones with the mitochondrial uncoupler, dinitrophenol (DNP) and determined the effect on ATP production and cell growth. We did not observe a decrease in ATP in the CD44+/MyD88+ EOC stem cells until after the 8h treatment with DNP (Fig. 5A). In fact, a trend of compensatory increase in ATP was observed, although it was not sustained through time. Interestingly, when followed for 24h, EOC stem cells did not lose viability but instead had enhanced proliferation rate when treated with DNP compared to Vehicle (Fig. 5B). We also determined the effect of the ATP synthase inhibitor, Oligomycin. Similarly, EOC stem cells did not show ATP loss when treated with Oligomycin (Fig. 5C) and although there was an initial decrease in proliferation, these cells did not lose viability in the presence of Oligomycin (Fig. 5D). Taken together with the demonstration that EOC stem cells cannot sustain ATP levels under glucose-limiting conditions, this shows that OXPHOS indeed does not contribute to ATP production in the EOC stem cells. In contrast, in the CD44-/MyD88- EOC cells, a decrease in ATP production is observed when these cells are treated with DNP or Oligomycin (Fig. 5A,C). In addition, prolonged treatment showed a decrease in viable cells in the presence of DNP or Oligomycin (Fig. 5B,D). These results suggest that differentiated EOC cells can generate ATP from both glycolysis and OXPHOS and more importantly that in contrast to the EOC stem cells, CD44-/MyD88- EOC cells require OXPHOS to sustain growth.

### IKKβ controls levels of Cox-I in ovarian cancer cells

**Figure 6:**
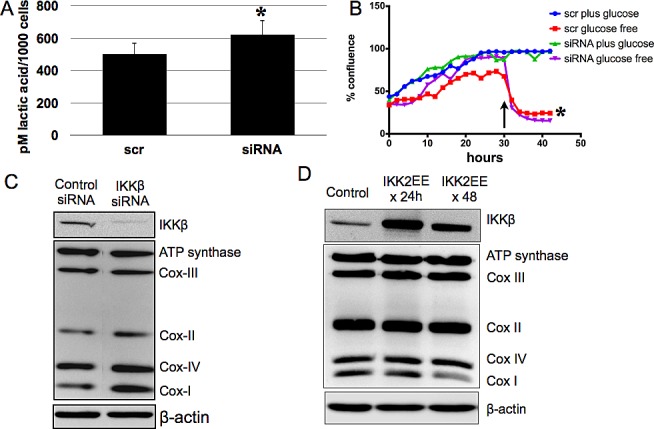
IKKβ does not fully control glycolytic phenotype of EOC stem cells but is inversely correlated with Cox-I CD44+/MyD88+ EOC stem cells were transfected with scramble siRNA (scr) or siRNA targeting IKKβ (siRNA). (A) Lactic acid was quantified as described above, (B) response to glucose deprivation was determined by measuring culture confluence using Incucyte, and (C) effect on components of electron transport chain determined by Western blot analysis. (D) A2780 cells were transfected with empty vector (Control) or pCMV-IKK2EE (IKK2EE) and levels of electron transport chain complexes were determined. * p > 0.05, not significant compared to scr. Arrow in B shows time of glucose deprivation.

We then sought to identify a specific pathway that may regulate the mitochondrial phenotype of the EOC stem cells. We previously showed that in contrast to the more differentiated CD44-/MyD88- EOC cells, CD44+/MyD88+ EOC stem cells are characterized by constitutive NFkB activity and constitutive secretion of pro-inflammatory cytokines brought in part by high levels of IKKβ [33, 34]. Previous studies have shown that NFκB can control the glycolytic phenotype through regulation of gene expression [35-37]. To determine if IKKβ/NFκB has any influence on the metabolic phenotype of the EOC stem cells, we performed transient transfection with IKKβ siRNA on the EOC stem cells. Transient knock-down of IKKβ did not result in changes in mitochondrial content nor MMP as detected by Mitotracker Green and Mitotacker Red, respectively (data not shown). IKKβ knock-down also did not result in any significant change in the levels of secreted lactic acid (Fig. 6A) and in addition, did not rescue the cells from death induced by glucose deprivation (Fig. 6B). However, molecular analysis showed a significant increase in the levels of Cox-I in the EOC stem cells with IKKβ siRNA compared to control/non-specific siRNA (Fig. 6C). We again demonstrated the inverse correlation between IKKβ and Cox-I by over-expressing constitutively active IKKβ (pCMV-IKK2EE) [33] in the classical ovarian cancer cell line, A2780. A2780 cultures are negative for CD44 and MyD88 and do not have constitutive NFkB activity [33]. We previously showed that ectopic expression of pCMV- IKK2EE in these cells is sufficient to induce secretion of pro-inflammatory cytokines such as IL-6, IL-8, MCP-1, and MIP1-α [33]. Interestingly, this also results in a decrease in Cox-I (Fig. 6D). Thus, IKKβ levels have an inverse correlation with Cox-I in ovarian cancer cells. However, IKKβ does not affect the other components of the electron transport chain. This may explain the similar response of control siRNA-transfected EOC stem cells and those with transient IKKβ knock-down to glucose deprivation. Taken together these results suggest that the bioenergetic phenotype of EOC stem cells is controlled at multiple levels and possibly regulated by multiple pathways.

### Maintenance with 2-DG delays recurrence

**Figure 7:**
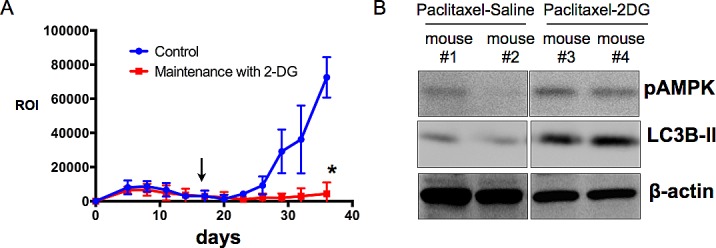
Maintenance with 2-DG decrease tumor burden in the recurrence setting (A) i.p. tumors were established with mCherry+ OCSC1-F2 cells as described in the Methods section. Paclitaxel was initiated on day 5 and ended on day 14. Maintenance treatment commenced on day 17 (arrow) and given 3 x a week. Tumor burden was monitored q3d for 36 days by live imaging and quantified by measuring region of interest (ROI) corresponding to mCherry fluorescence; (B) Tumors were analyzed for pAMPK and LC3B-II by Western blot analysis. * p = 0.003.

Our final objective is to test the translational application of our findings. In ovarian cancer, statistics show that primary disease have an excellent response rate to initial chemotherapy. However, as mentioned above, the problem arises from the development of chemoresistant recurrent disease. Since cancer stem cells represent the more chemoresistant population and are thought to contribute to recurrence, and since we show that glycolysis inhibitors are potent inducers of cancer stem cell death, a potential strategy is to follow chemotherapy, which targets differentiated cancer cells, with a maintenance regimen of glycolysis inhibitors, which can in turn target the remaining cancer stem cells. To test this hypothesis a “recurrent” ovarian cancer xenograft model is required. Our group recently described such a model. Intra-peritoneal ovarian tumors are established in nude mice and treated with Paclitaxel, which results in remission [38]. Cessation of chemotherapy at this point however, leads to the development of recurrent disease, which is resistant to second-round of Paclitaxel and thus mimics the clinical profile of ovarian cancer patients. Using this model, mice were treated with Paclitaxel and then randomly reassigned into 2 groups: maintenance with saline (Control) or maintenance with 2-DG. Our results show that mice maintained in 2-DG had slower progression to recurrence and decreased i.p. tumor burden compared to control group (Fig. 7A). Analysis of tumors obtained post-mortem recapitulates the biomarkers of response observed *in vitro*. Tumor lysates from mice maintained in 2-DG show upregulated p-AMPK and LC3B-II compared to mice maintained with saline (Fig. 7B). These results support the possible benefit of adding glycolysis inhibitors to standard chemotherapy with the goal of improving survival in ovarian cancer patients.

## DISCUSSION

We show in this study that in ovarian cancer, heterogeneity is not only exemplified by tumor-initiating potential or responsiveness to chemotherapy. There is likewise diversity in the bioenergetic requirements of the cancer cells that make up the tumor. The more chemoresponsive CD44-/MyD88- EOC cells require glucose for sustained proliferation but these differentiated cells can switch to OXPHOS under glucose limiting conditions. In addition, they can upregulate the glycolysis pathway if OXPHOS is inhibited demonstrating that the two routes for ATP synthesis are functional in these cells. In contrast, the inherently chemoresistant CD44+/MyD88+ EOC stem cells are solely reliant on glucose for survival since in these cells, multiple molecular blocks are present that prevent ATP production via OXPHOS. Consequently, since ATP can only be produced through glycolysis, inhibition of the glycolytic pathway becomes a potent route to induce cell death in these inherently chemoresistant cancer cell population. Since ovarian cancer mortality usually occurs in the recurrent setting and given the potential role of cancer stem cells in the promotion of recurrence, our study suggests the possible value of maintenance treatment with glycolysis inhibitors with the goal of improving patient survival.

Independence from OXPHOS can confer advantages. Cancer stem cells are classically defined as cancer cells that are able to self-renew and cause the different lineages of cancer cells that comprise the tumor [8, 10, 17, 18]. This definition requires the experimental proof of tumor formation in immune-incompetent mice. Since engraftment would require cells to survive at the site of injection (s.c. or i.p.), which can be hypoxic, independence from OXPHOS would therefore provide an advantage. In addition, independence from OXPHOS can support the process of sphere formation described in several cancer stem cell models. Indeed, we previously showed that the CD44+/MyD88+ EOC stem cells are able to undergo epithelial-mesenchymal transition (EMT) and mesenchymal-epithelial transition (MET) [39], which are processes that are indispensable in the generation of metastatic disease - a major contributor to ovarian cancer mortality. Hypoxia can potently initiate EMT in the CD44+/MyD88+ EOC stem cells resulting in the upregulation of the classical EMT inducer, Twist-1. Taken together, two possible advantages can be inferred from the observed bioenergetic phenotype of the EOC stem cells. First, the lower levels of ETC components suggest lower levels of oxygen requirement. Therefore, these cells may not be as sensitive to transient hypoxia. Second, the full reliance on glycolysis pathway for its ATP needs would assure stable ATP levels during engraftment or during the EMT-MET process, where oxygen may be extremely limited, but glucose may be obtained from intracellular glycogen stores.

Not all cancer cells exhibit the Warburg effect. In some cancer cells OXPHOS is the major source of ATP [40, 41] and in contrast to what was originally thought, recent studies have demonstrated that the mitochondria of some cancer cells are not damaged or mutated and are able to contribute to ATP generation [42]. These differences are probably reflections of the heterogeneity in cancer cell types as well as differences in cellular state. Indeed, Smolkova et al showed that controlled changes in gene expression during the process of tumorigenesis dynamically control OXPHOS activity [43]. This suggests that a cell type may have a preferential pathway to generate ATP under basal conditions but survival requires the capacity to engage the other route of ATP production under stress conditions. Our results show that this is not the case for EOC stem cells. These cells are not able to switch to OXPHOS when glucose levels are limiting and even when OXPHOS substrates are provided. The inability of CD44+/MyD88+ EOC stem cells to generate ATP through OXPHOS is regulated in at least three levels: PDH, UCP2, and the electron transport chain. Therefore, due to the low levels of PDH, pyruvate cannot be utilized by the Citric Acid cycle to generate reducing equivalents for the ETC. This leaves the conversion of pyruvate to lactic acid as the sole means to generate ATP. Even in conditions that would support an epigenetic switch leading to a gain of PDH, it can be inferred that the low baseline levels of ETC components can function as the second-level block since it may not be able to process the elevated levels of reducing equivalents and instead may generate significant amounts of reactive oxygen species. Finally, even when PDH and ETC are not limited, generation of ATP can be further inhibited in the EOC stem cells by UPC2, which diverts the proton motive force away from ATP synthase. It is important to note that the demonstration that EOC stem cells have higher levels of UCP2 yet also have higher MMP is not contradictory to what is currently known about UCP2. Previous studies have demonstrated UCP2 knock-down may further increase MMP [44] hence suggesting that UCP2 activity may be depend on cell type or cellular status.

The multiple levels of OXPHOS blockage enumerated above may explain why EOC stem cells cannot sustain ATP, and therefore undergo autophagic cell death when glucose levels are limiting. More importantly, these results suggest the value of glycolysis inhibitors in targeting these chemoresistant cancer cells. Since activation of alternative pathways is one of the major causes of targeted therapy failure *in vivo*, the demonstration that mitochondrial OXPHOS in EOC stem cells is blocked at multiple levels indicates that switching to OXPHOS when glucose is unavailable cannot be easily accomplished.

The identification of the most optimal treatment schedule is as important as finding an effective treatment modality. The Warburg effect has been described for decades but although its clinical impact has been useful in diagnostics as demonstrated by the usefulness of PET scans, its full translational effect in terms of therapies has not been fully realized. Taken together with statistical data that chemotherapy is effective in inducing remission in patients with primary disease and that ovarian cancer recurrence is driven at least in part by the CD44+/MyD88+ EOC stem cells that survive chemotherapy, the demonstration that EOC stem cells succumb to glycolysis inhibitors suggests that the most optimal treatment schedule is to give these inhibitors after the first-line chemotherapy. Indeed, we demonstrate the efficacy of 2-DG given as maintenance in the recurrence setting. 2-DG given after chemotherapy decreases tumor burden in the i.p. ovarian cancer xenograft model. It should also be noted that cancer cells in general succumb to glycolysis inhibitors more so than normal cells. Therefore the demonstration that EOC stem cells are even more avid towards glycolysis compared to the more differentiated cancer cells suggests a wider therapeutic window. This is extremely important since a narrow therapeutic window has been the major limiting factor in using chemotherapy to induce cell death in cancer stem cells.

Failure of specifically targeted treatments can be mostly explained by activation of alternative pathways that are redundant to the targeted pathway. This is especially true for kinase inhibitors wherein inhibition of one specific kinase deemed to be required for survival leads to the activation of alternative kinase/s consequently inhibiting efficacy of the treatment. In targeting bioenergetic pathways, the use of glycolysis inhibitors can be made ineffective by enhancing OXPHOS. Therefore the demonstration that the chemoresistant EOC stem cells are not able to utilize OXPHOS to generate ATP and that this is blocked by at least three independent mechanisms suggests the potential value of adding glycolysis inhibitors to conventional chemotherapy. This approach may lead to complete eradication of the tumor and may therefore improve survival in ovarian cancer patients.

## METHODS

### Cell cultures and conditions

OCSC1, OCSC2, OCSC5, OCSC6 (CD444+/MyD88+ EOC stem cell clones) were purified from patients diagnosed with serous EOC and thus each clone is from a different patient. OCC1 and OCC2 (CD44-/MyD88- EOC clones) are derived from OCSC1 and OCSC2, respectively by *in vitro* differentiation [20] and have similar characteristics as CD44-/MyD88- EOC cells isolated from primary tumors. OCC3 (CD44-/MyD88- EOC clone) was also obtained from a patient with serous EOC. Cells were isolated and cultured as previously described in these previous publications [20, 21, 33, 34, 38, 39, 45-48]. Purity of the EOC stem cell cultures based on CD44 expression (100% expression) was tested before each experiment by flow cytometry. Cells are never passed beyond 10 passages for any of the experiments. For each passage, in addition to CD44 levels, expression of MyD88 and other stemness associated markers previously described for these clones (including Oct-4 and Nanog) [20, 33, 45] are determined by western blot analysis and quantitative PCR. All sample collection described in this study were performed with patient consent and approved by the Human Investigation Committee of Yale University School of Medicine. High glucose Dulbecco's Modified Eagle Medium (Life Technologies, Grand Island, NY), with 25 mM of D-glucose was used to culture clones in glucose-enriched conditions. No glucose Dulbecco's Modified Eagle Medium (Life Technologies) was used to culture clones in glucose-free conditions.

### Reagents and treatment

2-deoxyglucose (2-DG) was purchased from Tocris Bioscience (Bristol, UK) and used at 20 mM. Dimethyl succinate was purchased from Sigma-Aldrich (St. Louis, MO) and used at 20 mM. Dinitrophenol was purchased from Sigma-Aldrich and used at 1 mM.

### Determination of cell growth, morphology, and viability

Growth curves and cellular morphology were assessed using Incucyte (Essen Instruments, Ann Arbor, MI), a kinetic live cell imaging system. Proliferation was measured through quantitative kinetic processing metrics derived from time-lapse image acquisition and presented as percentage of culture confluence over time. Effect of treatment on cell viability was quantified using Celltiter^96^ Aqueous One Solution Proliferation Assay (Promega, Madison, WI).

### Caspase activity assay

Total protein was extracted and measured as previously described [46, 49]. Activity of caspase 3/7 and caspase 9 was quantified using Caspase Glo 3/7 and Caspase Glo 9, respectively (Promega) according to manufacturer's instructions. Positive control for caspase activation is lysate from the ovarian cancer cell line, A2780 treated for 24h with 100 μg/ml carboplatin.

### Western blot analysis

SDS-PAGE and Western blots were performed using 20 ug of total protein lysate as previously described [46, 49]. Antibodies used were: rabbit anti-LC3B (Cell Signaling Technology, Danvers, MA), rabbit anti-phospho AMPK (Cell Signaling Technology, Danvers, MA), rabbit anti-actin (Sigma Aldrich, St. Louis, MI), Mitoprofile Total OXPHOS Human WB antibody cocktail (Abcam, Cambridge, MA), rabbit anti-pyruvate dehyrogenase (Cell Signaling Technology), rabbit anti phospho-pyruvate dehyrogenase E1 (S293) (Abcam) and anti-rabbit UCP2 (Abcam).

### Quantification of ATP

ATP was quantified from live cells using CellTiter-Glo Luminescent Assay (Promega) according to manufacturer's instructions. Data was normalized to cell number.

### Quantification of lactic acid

Lactic acid was quantified from cell-free culture supernatants using Lactate Colorimetric Assay Kit II (Biovision, Inc.. Milpitas, CA) according to manufacturer's instructions. Data was normalized to cell number.

### Determination of mitochondrial mass and mitochondrial membrane potential

Mitochondrial mass and mitochondrial membrane potential were determined by flow cytometry using Mitotracker Green FM (Invitrogen, Carlsbad, CA) and Mitotracker Red CMXRos (Molecular Probes) as previously described [21]. Flow cytometry data were acquired using BD FACSCalibur and analyzed using CellQuest (BD Biosciences, San Jose, CA).

### IKKβ transfection and knockdown

Transient transfection with pCMV-IKK2EE was carried out using XtremeGENE 9 DNA Transfection Reagent (Roche Applied Bioscience) as previously described. siRNA specifically targeting IKKβ and a control scramble siRNA was purchased from Life Technologies (Grand Island, NY) and transfected into EOC stem cells using siPORT (Life Technologies) according to manufacturer's instructions.

### Recurrent ovarian cancer xenograft model, treatment schedule, and *in vivo* imaging

The Yale University Institutional Animal Care and Use Committee approved all *in vivo* studies described. The intraperitoneal (i.p) recurrent ovarian cancer xenograft model was established as previously described [38, 50] using OCSC1-F2 cells stably expressing the mCherry fluorescent tag. Paclitaxel was given i.p. at 12 mg/kg q3d and afterwards mice were randmomized into 2 groups: (1) Maintenance with saline as control, n = 10 and maintenance with 2-DG, n= 4. 2-DG was given at 500 mg/kg i.p. qd. Tumor growth was monitored by live *in vivo* animal imaging using FX PRO (Bruker Corp., Billerica, MA) and using mCherry fluorescence as surrogate for tumor burden.

### Statistical analysis

Data are graphed and analyzed using Prism 6 (GraphPad Software, Inc.). Unpaired t test is used to compare one variable and assumes that populations have the same variances or standard deviation. Area under the curve (AUC) analysis is used to allow the cumulative measurement of effect through time instead of one single time-point. p values < 0.05 are considered significant.

## References

[1] Rooth C (2013). Ovarian cancer: risk factors, treatment and management. British journal of nursing.

[2] Siegel R, Naishadham D, Jemal A (2012). Cancer statistics, 2012. CA Cancer J Clin.

[3] Wright JD, Neugut AI, Lewin SN, Lu YS, Herzog TJ, Hershman DL (2013). Trends in hospital volume and patterns of referral for women with gynecologic cancers. Obstetrics and gynecology.

[4] Jelovac D, Armstrong DK (2011). Recent progress in the diagnosis and treatment of ovarian cancer. CA Cancer J Clin.

[5] Covens A, Carey M, Bryson P, Verma S, Fung Kee Fung M, Johnston M (2002). Systematic review of first-line chemotherapy for newly diagnosed postoperative patients with stage II, III, or IV epithelial ovarian cancer. Gynecologic oncology.

[6] du Bois A, Luck HJ, Meier W, Adams HP, Mobus V, Costa S, Bauknecht T, Richter B, Warm M, Schroder W, Olbricht S, Nitz U, Jackisch C, Emons G, Wagner U, Kuhn W (2003). A randomized clinical trial of cisplatin/paclitaxel versus carboplatin/paclitaxel as first-line treatment of ovarian cancer. J Natl Cancer Inst.

[7] Ozols RF (1995). Combination regimens of paclitaxel and the platinum drugs as first-line regimens for ovarian cancer. Semin Oncol.

[8] Clarke MF, Dick JE, Dirks PB, Eaves CJ, Jamieson CH, Jones DL, Visvader J, Weissman IL, Wahl GM (2006). Cancer stem cells--perspectives on current status and future directions: AACR Workshop on cancer stem cells. Cancer Res.

[9] Clarke MF, Fuller M (2006). Stem cells and cancer: two faces of eve. Cell.

[10] Dalerba P, Cho RW, Clarke MF (2007). Cancer stem cells: models and concepts. Annu Rev Med.

[11] Dean M, Fojo T, Bates S (2005). Tumour stem cells and drug resistance. Nat Rev Cancer.

[12] Giuffrida D, Rogers IM (2010). Targeting cancer stem cell lines as a new treatment of human cancer. Recent patents on anti-cancer drug discovery.

[13] Hemmings C (2010). The elaboration of a critical framework for understanding cancer: the cancer stem cell hypothesis. Pathology.

[14] Huang EH, Heidt DG, Li CW, Simeone DM (2007). Cancer stem cells: a new paradigm for understanding tumor progression and therapeutic resistance. Surgery.

[15] Nie D (2010). Cancer stem cell and niche. Frontiers in bioscience.

[16] Sell S (2010). On the stem cell origin of cancer. The American journal of pathology.

[17] Winquist RJ, Furey BF, Boucher DM (2010). Cancer stem cells as the relevant biomass for drug discovery. Current opinion in pharmacology.

[18] Reya T, Morrison SJ, Clarke MF, Weissman IL (2001). Stem cells, cancer, and cancer stem cells. Nature.

[19] Morrison R, Schleicher SM, Sun Y, Niermann KJ, Kim S, Spratt DE, Chung CH, Lu B (2011). Targeting the mechanisms of resistance to chemotherapy and radiotherapy with the cancer stem cell hypothesis. J Oncol.

[20] Alvero AB, Chen R, Fu HH, Montagna M, Schwartz PE, Rutherford T, Silasi DA, Steffensen KD, Waldstrom M, Visintin I, Mor G (2009). Molecular phenotyping of human ovarian cancer stem cells unravels the mechanisms for repair and chemoresistance. Cell Cycle.

[21] Alvero AB, Montagna MK, Holmberg JC, Craveiro V, Brown D, Mor G Targeting the mitochondria activates two independent cell death pathways in ovarian cancer stem cells. Mol Cancer Ther.

[22] Bapat SA (2010). Human ovarian cancer stem cells. Reproduction.

[23] Casagrande F, Cocco E, Bellone S, Richter CE, Bellone M, Todeschini P, Siegel E, Varughese J, Arin-Silasi D, Azodi M, Rutherford TJ, Pecorelli S, Schwartz PE, Santin AD (2011). Eradication of chemotherapy-resistant CD44+ human ovarian cancer stem cells in mice by intraperitoneal administration of Clostridium perfringens enterotoxin. Cancer.

[24] Chefetz I, Holmberg JC, Alvero AB, Visintin I, Mor G Inhibition of Aurora-A kinase induces cell cycle arrest in epithelial ovarian cancer stem cells by affecting NFkB pathway. Cell Cycle.

[25] Liu M, Mor G, Cheng H, Xiang X, Hui P, Rutherford T, Yin G, Rimm DL, Holmberg J, Alvero A, Silasi DA (2013). High frequency of putative ovarian cancer stem cells with CD44/CK19 coexpression is associated with decreased progression-free intervals in patients with recurrent epithelial ovarian cancer. Reprod Sci.

[26] Steffensen KD, Alvero AB, Yang Y, Waldstrom M, Hui P, Holmberg JC, Silasi DA, Jakobsen A, Rutherford T, Mor G (2011). Prevalence of epithelial ovarian cancer stem cells correlates with recurrence in early-stage ovarian cancer. J Oncol.

[27] Warburg O (1956). On respiratory impairment in cancer cells. Science.

[28] Warburg O, Wind F, Negelein E (1927). The Metabolism of Tumors in the Body. The Journal of general physiology.

[29] Gatenby RA, Gillies RJ (2004). Why do cancers have high aerobic glycolysis?. Nat Rev Cancer.

[30] Jose C, Bellance N, Rossignol R (2011). Choosing between glycolysis and oxidative phosphorylation: a tumor's dilemma?. Biochimica et biophysica acta.

[31] Riganti C, Gazzano E, Polimeni M, Aldieri E, Ghigo D (2012). The pentose phosphate pathway: an antioxidant defense and a crossroad in tumor cell fate. Free Radic Biol Med.

[32] Pedersen PL (1978). Tumor mitochondria and the bioenergetics of cancer cells. Progress in experimental tumor research.

[33] Chen R, Alvero AB, Silasi DA, Kelly MG, Fest S, Visintin I, Leiser A, Schwartz PE, Rutherford T, Mor G (2008). Regulation of IKKbeta by miR-199a affects NF-kappaB activity in ovarian cancer cells. Oncogene.

[34] Leizer AL, Alvero AB, Fu HH, Holmberg JC, Cheng YC, Silasi DA, Rutherford T, Mor G Regulation of inflammation by the NF-kappaB pathway in ovarian cancer stem cells. Am J Reprod Immunol.

[35] Mauro C, Leow SC, Anso E, Rocha S, Thotakura AK, Tornatore L, Moretti M, De Smaele E, Beg AA, Tergaonkar V, Chandel NS, Franzoso G (2011). NF-kappaB controls energy homeostasis and metabolic adaptation by upregulating mitochondrial respiration. Nature cell biology.

[36] Johnson RF, Perkins ND (2012). Nuclear factor-kappaB, p53, and mitochondria: regulation of cellular metabolism and the Warburg effect. Trends in biochemical sciences.

[37] Johnson RF, Witzel II, Perkins ND (2011). p53-dependent regulation of mitochondrial energy production by the RelA subunit of NF-kappaB. Cancer Res.

[38] Craveiro V, Yang-Hartwich Y, Holmberg J, Sumi N, Pizzonia J, Griffin B, Gill S, Silasi D, Azodi M, Rutherford T, Alvero A.B, Mor G (2013). Phenotypic Modifications in Ovarian Cancer Stem Cells Following Paclitaxel Treatment. Cancer Medicine.

[39] Yin G, Alvero AB, Craveiro V, Holmberg JC, Fu HH, Montagna MK, Yang Y, Chefetz-Menaker I, Nuti S, Rossi M, Silasi DA, Rutherford T, Mor G (2013). Constitutive proteasomal degradation of TWIST-1 in epithelial-ovarian cancer stem cells impacts differentiation and metastatic potential. Oncogene.

[40] Reitzer LJ, Wice BM, Kennell D (1979). Evidence that glutamine, not sugar, is the major energy source for cultured HeLa cells. J Biol Chem.

[41] Rodriguez-Enriquez S, Carreno-Fuentes L, Gallardo-Perez JC, Saavedra E, Quezada H, Vega A, Marin-Hernandez A, Olin-Sandoval V, Torres-Marquez ME, Moreno-Sanchez R (2010). Oxidative phosphorylation is impaired by prolonged hypoxia in breast and possibly in cervix carcinoma. The international journal of biochemistry & cell biology.

[42] Bellance N, Benard G, Furt F, Begueret H, Smolkova K, Passerieux E, Delage JP, Baste JM, Moreau P, Rossignol R (2009). Bioenergetics of lung tumors: alteration of mitochondrial biogenesis and respiratory capacity. The international journal of biochemistry & cell biology.

[43] Smolkova K, Plecita-Hlavata L, Bellance N, Benard G, Rossignol R, Jezek P (2011). Waves of gene regulation suppress and then restore oxidative phosphorylation in cancer cells. The international journal of biochemistry & cell biology.

[44] Sayeed A, Meng Z, Luciani G, Chen LC, Bennington JL, Dairkee SH (2010). Negative regulation of UCP2 by TGFbeta signaling characterizes low and intermediate-grade primary breast cancer. Cell death & disease.

[45] Alvero AB, Fu HH, Holmberg J, Visintin I, Mor L, Marquina CC, Oidtman J, Silasi DA, Mor G (2009). Stem-like ovarian cancer cells can serve as tumor vascular progenitors. Stem Cells.

[46] Alvero AB, Montagna MK, Chen R, Kim KH, Kyungjin K, Visintin I, Fu HH, Brown D, Mor G (2009). NV-128, a novel isoflavone derivative, induces caspase-independent cell death through the Akt/mammalian target of rapamycin pathway. Cancer.

[47] Alvero AB, Montagna MK, Craveiro V, Liu L, Mor G Distinct subpopulations of epithelial ovarian cancer cells can differentially induce macrophages and T regulatory cells toward a pro-tumor phenotype. Am J Reprod Immunol.

[48] Chefetz I, Alvero AB, Holmberg JC, Lebowitz N, Craveiro V, Yang-Hartwich Y, Yin G, Squillace L, Gurrea Soteras M, Aldo P, Mor G (2013). TLR2 enhances ovarian cancer stem cell self-renewal and promotes tumor repair and recurrence. Cell Cycle.

[49] Alvero AB, O'Malley D, Brown D, Kelly G, Garg M, Chen W, Rutherford T, Mor G (2006). Molecular mechanism of phenoxodiol-induced apoptosis in ovarian carcinoma cells. Cancer.

[50] Pizzonia J, Holmberg J, Orton S, Alvero A, Viteri O, McLaughlin W, Feke G, Mor G (2012). Multimodality animal rotation imaging system (Mars) for *in vivo* detection of intraperitoneal tumors. Am J Reprod Immunol.

